# Editorial: Acute kidney injury: from pathology to phytotherapy

**DOI:** 10.3389/fphar.2025.1649837

**Published:** 2025-07-17

**Authors:** Ya-Long Feng, Wan-Ying Ma, Yin-Xuan Jiang, Le Shui, Hua Chen, Xue-Zhong Gong

**Affiliations:** ^1^ Department of Life Science, Xianyang Normal University, Xianyang, Shaanxi, China; ^2^ College of Pharmacy, Ningxia Medical University, Yinchuan, Ningxia, China; ^3^ Department of Nephrology, Shanghai Municipal Hospital of Traditional Chinese Medicine, Shanghai University of Traditional Chinese Medicine, Shanghai, China

**Keywords:** acute kidney injury, traditional medical herb, pathology, phytotherapy, natural product

## Introduction

Acute kidney injury (AKI), defined by an abrupt rise in serum creatinine and a decrease in urinary output, causes a high morbidity and mortality worldwide (Ronco et al., 2019). Due to the poor understanding of AKI pathophysiology, effective drugs for AKI treatment remain lacking. Therefore, the cellular and molecular mechanisms of AKI should be deeply studied, which will be beneficial for developing therapy drugs against AKI.

Traditional medical herb has been used to treat AKI for many years. A large number of compounds have been isolated and identified from traditional medical herbs. Recent studies reveal that some of these compounds can alleviate AKI by inhibiting inflammation, oxidative stress, and apoptosis. Noteworthily, some compounds exhibited their therapeutic efficacy on AKI in clinics, such as anisodamine, indicating an important source of traditional medical herbs in drug discovery for AKI. However, the active ingredients and mechanisms of traditional medical herbs against AKI are still poorly understood.

The present Research Topic aims at collecting the manuscripts demonstrating the novel findings in the pathological process of AKI and the protective effects of compounds derived from traditional medical herbs against AKI. After rigorous peer reviews, a total of 16 manuscripts were published.

## The biomarker for AKI diagnosis

The early diagnosis and intervention are very important for AKI treatment. Ryu et al. found that the urinary L-liver-type fatty acid-binding protein (L-FABP) level had a close relationship with AKI progression (Yasuda et al.). Further study revealed that the L-FABP level should be measured at 6 and 12 h after kidney injury than only one time at 6 h, suggesting a more precise diagnose method for AKI. In addition, Gong et al. found that the serum levels of human epididymis protein 4 (HE4) and N-terminal pro-B-type natriuretic peptide (NT-proBNP) were increased in patients with chronic kidney diseases (CKD) and patients with AKI on CKD (Song et al.). The level of HE4 is positively correlated with the disease severity, and patients with higher levels of HE4 and NT-proBNP usually have poorer prognosis. These results suggest that the serum levels of HE4 and NT-proBNP are impactful predictors of AKI on CKD.

## The cellular and molecular mechanisms of AKI

Chen et al. conducted a comprehensive bibliometric analysis of AKI and immune-related studies from the past two decades, identifying COVID-19, immune checkpoint inhibitors, regulated necrosis, cirrhosis-related AKI, and other emerging Research Topic as key research foci in recent years (Chen et al.).

Proprotein convertase subtilisin/kexin type 9 (PCSK9) is mainly expressed in liver and regulates cholesterol metabolism. The PCSK9 inhibitors are lipid-lowering drugs. Liu conducted a disproportionality analysis through the Food and Drug Administration Adverse Event Reporting System database and found that the treatment with PCSK9 inhibitors might induce AKI (Liu). In addition, vancomycin had significant nephrotoxicity. Pan et al. found that the incidence of vancomycin-associated acute kidney injury (VA-AKI) in patients with infective endocarditis (IE) was slightly higher than in general adult patients through conducting a large retrospective cohort study (Kunming et al.). It is worth noting that the combination of vancomycin with contrast agents significantly increased the nephrotoxic in patients with IE. In addition, vancomycin therapy longer than 10.75 days increased the risk of AKI. These results are beneficial for preventing AKI.

The NF-κB signaling pathway plays a key role in inflammation and oxidative stress. Ren et al. reviewed the important role of NF-κB signaling pathway in AKI, which provided a new insight to the treatment of AKI by targeting the NF-κB signaling pathway (Ren et al.). In addition, arachidonic acid (AA) is a main component of cell membrane lipids and is associated with kidney function. Li et al. reviewed the main metabolic pathways of AA in inflammation and oxidative stress during the processes of AKI, diabetic nephropathy and renal cell carcinoma (Li et al.).

Calprotectin (S100A8/A9) is crucial for leukocyte recruitment and inflammatory response. Shi et al. found that the S100A9 expression was increased continuously in AKI mice (Shi et al.). The S100A9 inhibitor, paquinimod, significantly improved renal function by inhibiting renal tubular epithelial cell apoptosis, inflammation, superoxide production, and mitochondrial dynamic imbalance, which suggested that S100A9 was a therapeutic target for treating AKI. In addition, apolipoprotein M (apoM) plays a key role in the reabsorption function of the kidney. Bisgaard et al. found that apoM was detectable in plasma of kidney-specific human transgenic mice and was secreted to both the apical (urine) and basolateral (blood) compartment from proximal tubular epithelial cells in HK-2 cells-overexpressed human apoM, suggesting a crucial role of apoM in sequestering molecules from excretion in urine (Bisgaard et al.). However, the overexpression of apoM did not protect against AKI.

Pyruvate kinase M2 (PKM2) is a rate-limiting enzyme in glycolysis. Chen et al. highlighted its critical roles in of kidney disease progression, including podocyte injury, fibroblast activation and proliferation, macrophage polarization, and T cell regulation (Chen et al.). Notably, both the activators and inhibitors of PKM2 showed a therapeutic effect in kidney diseases, underscoring their potential as AKI treatment strategies.

## Traditional medical herbs against AKI

Huangqi-Danshen decoction (HDD) is a well-known Chinese herbal preparation and shows a reno-protective effect. Liu et al. found that HDD protected against AKI by suppressing apoptosis, inflammation and oxidative stress via modulating the NAD^+^ biosynthesis (Liu et al.). *Panax notoginseng* is used to treat haemorrhage, blood-stasis, swelling and pain in China. Li et al. found that *Panax notoginseng* rhizomes (PNR) could decrease serum creatinine and urea nitrogen levels, reduce renal infarct areas and renal tubular cell injury areas, and inhibit renal cell apoptosis via the downregulation of MMP9, TP53 and IL-6 in mice with ischemia and reperfusion injury (Li et al.). *Prunella vulgari*s has a long history for treating kidney diseases in China. Yang et al. found that the main bio-active ingredients of *Prunella vulgari*s against COVID-19-associated AKI were quercetin, luteolin and kaempferol through the network pharmacology and molecular docking analysis (Yang et al.). The inhibition of NF-κB signaling pathway might be the key mechanism of *Prunella vulgari*s against AKI. *Rhizoma Chuanxiong* (CX) and *Radix et Rhizoma Rhei* (DH) are well known traditional Chinese medicines. Li et al. found that the core bio-active ingredients of CX and DH included aloe-emodin, (-)-catechin, β-sitosterol and folic acid though network pharmacology method (Li et al.). In contrast media-induced AKI rats, CX and DH alleviated AKI by regulating inflammation, cell death and cell cycle processes via inhibiting the p38-MAPK/p53 signaling pathway. Yue-bi-tang (YBT) is a traditional formula used to treat edema. Li et al. found that YBT alleviated edema by decreasing renal microvascular permeability via suppressing the Cav-1/eNOS signaling pathway in Adriamycin-treated rats (Li et al.).

Myricitrin is a natural flavonoid compound with diverse pharmacological properties, such as anti-oxidant, anti-inflammation and anti-cancer activities. Huang et al. found that the pretreatment of myricitrin significantly rescued HK-2 cells from cell death and reduced iron overload (Lin et al.). The protective effect of myricitrin could be reserved by the inducer of ferritinophagy rapamycin, which might suggest that myricitrin ameliorated kidney injury by attenuating ferritinophagy-mediated ferroptosis.

## Conclusion

The Research Topic “Acute Kidney Injury: From Pathology to Phytotherapy” has collected worthy studies and contributions on the subject of AKI, highlighting the promising therapeutic property of traditional medical herb. We hope that you enjoy and gain from the Research Topic, which will surely promote the drug development for AKI and the further application of traditional medical herb.
